# Neuromagnetic Evidence for Early Auditory Restoration of Fundamental Pitch

**DOI:** 10.1371/journal.pone.0002900

**Published:** 2008-08-06

**Authors:** Philip J. Monahan, Kevin de Souza, William J. Idsardi

**Affiliations:** 1 Department of Linguistics, University of Maryland, College Park, Maryland, United States of America; 2 Neuroscience and Cognitive Science Program, University of Maryland, College Park, Maryland, United States of America; Victoria University of Wellington, New Zealand

## Abstract

**Background:**

Understanding the time course of how listeners reconstruct a missing fundamental component in an auditory stimulus remains elusive. We report MEG evidence that the missing fundamental component of a complex auditory stimulus is recovered in auditory cortex within 100 ms post stimulus onset.

**Methodology:**

Two outside tones of four-tone complex stimuli were held constant (1200 Hz and 2400 Hz), while two inside tones were systematically modulated (between 1300 Hz and 2300 Hz), such that the restored fundamental (also knows as “virtual pitch”) changed from 100 Hz to 600 Hz. Constructing the auditory stimuli in this manner controls for a number of spectral properties known to modulate the neuromagnetic signal. The tone complex stimuli only diverged on the value of the missing fundamental component.

**Principal Findings:**

We compared the M100 latencies of these tone complexes to the M100 latencies elicited by their respective pure tone (spectral pitch) counterparts. The M100 latencies for the tone complexes matched their pure sinusoid counterparts, while also replicating the M100 temporal latency response curve found in previous studies.

**Conclusions:**

Our findings suggest that listeners are reconstructing the inferred pitch by roughly 100 ms after stimulus onset and are consistent with previous electrophysiological research suggesting that the inferential pitch is perceived in early auditory cortex.

## Introduction

Pitch is the perceptual correlate of the fundamental periodic component of an auditory signal (F_0_). An accurate encoding of the information carried in the fundamental component is required for the successful perception of various kinds of linguistic and paralinguistic information (e.g., lexical tone, intonation, voicing, and speaker identification and emotional state) and non-linguistic auditory input (e.g., music perception). Listeners are adept, however, at recovering the fundamental component from alternative regions of frequency space when the fundamental component itself is missing or masked [1]–[4]. One everyday example of this effect can be observed with adult voices transmitted telephonically: the fundamental component of the voice is typically below 300 Hz, but narrowband digital telephony transmits only between 300–3400 Hz. Consequently, the listener must *reconstruct* the pitch from the signal in the passband. Given the relative importance of its contribution, recovering the pitch of a signal is integral for constructing a holistic percept for a given auditory stimulus and ultimately arriving at the recognition of an auditory object. The present study uses magnetoencephalography (MEG) to measure an early, automatic evoked auditory response, the M100 (or N1m), building on and extending some previous studies that required some clarification. We find that the M100 latencies of the inferred pitch stimuli match those evoked by actual sinusoidal tones with the same frequencies, suggesting that inferred pitch is recovered by 100 ms and, moreover, that the M100 encodes computations performed over the input and not just transparent spectral properties of the stimulus.

The neural mechanisms that reconstruct the lower end of the frequency spectrum and reconstitute information present in the fundamental component are still largely unknown (see [5], [6] for models). Listeners' ability to reconstruct this spectral information, and in particular, to recover the fundamental component (F_0_), has been termed fundamental restoration, also known as *inferred pitch*, the *missing fundamental* phenomenon or *virtual pitch* [6]. This phenomenon has also been observed in non-human mammals [7]–[10]. From a neurophysiological perspective, understanding the time course of fundamental restoration is a prerequisite to identifying the range of neurobiological mechanisms potentially responsible for the reconstruction of the fundamental component.

Recently, the temporal and spatial dynamics of fundamental restoration have been explored using electrophysiology [11]–[14]. The focus of this work has been on determining the neuroanatomical basis of fundamental restoration. In particular, by employing source-localization analysis of the M100, the fundamental restoration has been localized to the transverse temporal gyrus and the superior temporal gyrus [13]. Moreover, independent neural generators appear to underlie the perception of pure sinusoids and their inferred fundamental counterparts [11]. In an attempt to understand the temporal dynamics of fundamental restoration, Winkler and colleagues found no latency or amplitude differences using EEG in the N1 between spectral and restored fundamental stimuli [14]. The only differences they found were to tokens with long durations (500 ms, as opposed to 150 ms in duration) in a mismatch negativity paradigm.

Perhaps most notably, Pantev and colleagues used MEG and compared the neuromagnetic responses to two sinusoids (250 Hz and 1000 Hz) and a tone complex with an inferred pitch of 250 Hz (1000 Hz, 1250 Hz, 1500 Hz and 1750 Hz) [12]. Presenting a source-based analysis of the MEG responses, they concluded that the neural generators of the M100 reflect the processing of the subjective perception of the pitch of a stimulus and not the actual stimulus properties. In other words, the neuronal computations required to reconstruct the fundamental component are performed within 100 ms post onset of the target and reside in early auditory cortex. While the evidence we present here is consistent with this conclusion, there are some caveats that should be noted regarding their findings. First, for the tone complex used in their study, they inserted a continuous band-pass noise centered at 250 Hz, essentially building an equivalent actual pitch into the stimulus that was intended to elicit an inferred pitch. The findings would have been much more convincing had they used a broader band of noise as a spectral masker, say from DC to 500 Hz. Second, the sampling rate for the early MEG equipment was coarse (250 Hz), thereby making it difficult to assign an interpretation to the latency data. The reported latency differences were 4 ms, or one sample at this sampling rate.

Independent research on the M100 suggests that its latency is modulated by spectral characteristics of auditory input. In particular, M100 response latencies are shortest to sinusoids with a frequency of 1000 Hz and longer to frequencies both above and below 1000 Hz (i.e., forming a parabola centered near 1000 Hz) [15]. Therefore, if the neuromagnetic signal was, indeed, primarily reflecting the reconstruction of a fundamental component, then we should expect the latencies for the 250 Hz sinusoid and the tone complex with a 250 Hz inferred pitch to have roughly the same latency, and both should be significantly longer than the M100 response to the 1000 Hz sinusoid. This straightforward prediction is only borne out in two of the six participants reported in the Pantev study [12]. In a more recent electrophysiological study investigating the neurobiological properties of fundamental restoration, Fujioka and colleagues [11] compared neuromagnetic responses to tone complexes with inferred fundamentals of 250 Hz, 500 Hz and 1000 Hz composed of their 2^nd^ through 5^th^ harmonics, 6^th^ through 9^th^ harmonics and 10^th^ through 13^th^ harmonics. They report that all stimulus parameters (periodicity, harmonic order level, stimulus type (pure tone, inferred fundamental inducing tone complex)) affected M100 latency.

It is also known that the M100 response latency is sensitive to the spectral center of gravity of auditory stimuli [16]. In the Fujioka et al. study, however, unfortunately the conditions are confounded, and therefore any differences in auditory evoked latencies could be attributed to significant differences in the spectral center of gravity. Therefore, to control for differences in the spectral center of gravity, while systematically modulating the induced fundamental component, we synthesized sinusoidal tone complexes with side bands that were kept constant across the different tokens (1200 Hz and 2400 Hz) and two additional sinusoids within these sidebands. This allowed us to systematically control the spectral center of gravity, while the internal sinusoids contributed the frequency of the inferred fundamental.

## Materials and Methods

### Subjects

Nine (7 female; age range = 20–59; mean age = 26.3) healthy, right-handed adult volunteers with normal hearing participated in this study. All tested strongly right-handed on the Edinburgh Handedness Survey [17] and were compensated $10/hr for their participation. Each session lasted approximately 1½ to 2 hours. Participants provided written informed consent. The involvement of human participants in the reported experiment was approved by the University of Maryland, College Park Institutional Review Board (IRB).

### Stimuli

Two different sets of auditory stimuli were synthesized using Praat [18] at a sampling frequency of 44.1 KHz. Each stimulus was 70 ms in duration with 10 ms linear rise and decay ramps. The first set were pure sinusoids at 100 Hz, 200 Hz, 300 Hz, 400 Hz, 600 Hz, 1200 Hz and 2400 Hz. The second set of stimuli consisted of sinusoidal complexes. Each complex was composed of up to four component sinusoids. Two of the four sinusoids for all tone complexes were shoulder tones at 1200 Hz and 2400 Hz; the two other sinusoids were placed between the shoulder tones. The frequency of these two internal sinusoids varied to produce inferred fundamentals corresponding to the frequencies of the pure tone sinusoids. For example, the tone complex with an inferred fundamental component of 400 Hz was composed of equal amplitude sinusoids at 1200 Hz, 1600 Hz, 2000 Hz and 2400 Hz. One additional complex contained only the shoulder tones (i.e., 1200 Hz and 2400 Hz). The amplitudes of the sounds were chosen as a compromise between matching the physical sound level and the psychophysical intensity (i.e., from a hearing threshold curve). The complex stimuli had an average intensity of 84 dB SPL, and the pure sinusoids had an average intensity of 90 dB SPL, these values appeared to be relatively well-matched for listeners.

**Figure 1:**
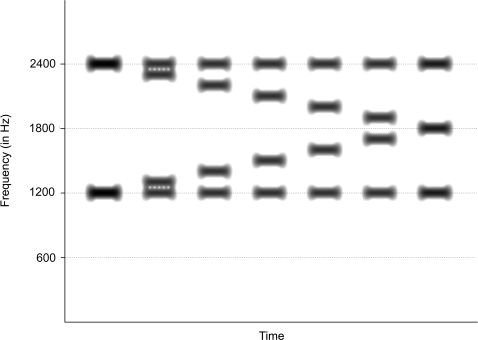
A composite spectrogram of the seven complex tones used in the experiment. The duration of each complex tone was 70 ms, including 10 ms rise and decay time. Each complex tone included shoulder tones of 1200 Hz and 2400 Hz. Internal sidebands were synthesized in 100 Hz steps inward from the shoulder tones in six of the seven stimuli to induce the inferred fundamental components.

The particular nature of the structure of the tone complexes is important. First, by placing shoulder tones at 1200 Hz and 2400 Hz and successively moving the internal tones closer to the midpoint (i.e., 1800 Hz) in 100 Hz steps, we ensured that the spectral center of gravity (the first spectral moment, M_1_) would remain constant across the tone complexes. This is evident in Table 1, where it is shown that the spectral center of gravity, M_1_, is 1800 Hz across all tone complexes. Again, this is important given that the latency of the M100 has been found to be sensitive to this property of the stimulus [16], a potential confound in some of the previous electrophysiological studies on the perception of the inferred fundamental (e.g., [11]). Constructing the sinusoidal complexes in this manner also controls for skewness (the third moment, M_3_) and kurtosis (the fourth moment, M_4_). Thus, we can be confident in attributing the response profile of the M100 of these tone complexes solely to the inferred fundamental and not to some overall spectral shape property of the stimuli. Figure 1 presents a spectrogram showing all seven four tone complexes.

**Table 1:** Spectral values of the auditory stimuli.

F_Inf_	F_1_	F_2_	F_3_	F_4_	M_1_	M_2_	M_3_	M_4_
Pure sinusoids								
	100				100	8.82	0.82	37
	200				200	9.14	0.78	65
	300				300	9.24	0.76	94
	400				400	9.30	0.75	123
	600				600	9.35	0.74	179
	1200				1200	9.40	0.73	337
	2400				2400	9.43	0.72	606
Tone Complexes								
100	1200	1300	2300	2400	1800	552	−0.000020	−1.97
100	1200	1700	1900	2400	1800	430	0.000020	−1.10
200	1200	1400	2200	2400	1800	510	−0.000010	−1.85
300	1200	1500	2100	2400	1800	474	−0.000002	−1.64
400	1200	1600	2000	2400	1800	447	0.000009	−1.36
600	1200	1800	1800	2400	1800	490	−0.000002	−1.50
1200	1200			2400	1800	600	−0.000002	−2.00

F_Inf_ = Inferred Fundamental (in Hz); F_1_ = First Harmonic (in Hz); F_2_ = Second Harmonic (in Hz); F_3_ = Third Harmonic (in Hz); F_4_ = Fourth Harmonic (in Hz); M_1_ = Spectral Centre of Gravity (in Hz); M_2_ = Standard Deviation (in Hz); M_3_ = Skewness; M_4_ = Kurtosis.

### Procedure

Magnetoencephalographic recordings were made using a 157-channel whole-head axial gradiometer MEG system (Kanazawa Institute of Technology, Kanazawa, Japan). Participants lay supine in a magnetically shielded room. Auditory stimuli were delivered binaurally via Etymotic ER3A insert earphones. Earphones were calibrated to have a flat frequency response between 50 Hz and 3100 Hz within the shielded room. The inter-stimulus interval (ISI) varied pseudo-randomly between 700 ms and 1500 ms. All auditory stimuli were presented 150 times each. Stimulus-related epochs of 700 ms (200 ms pre-trigger) were averaged according to stimulus type to improve the signal-to-noise ratio. The neuromagnetic signal was sampled at 1 KHz with an online 200 Hz LPF and 60 Hz notch filter. Offline, the data were noise reduced using a multi-shift PCA noise reduction algorithm [19] and was band-pass filtered by a Hamming-window digital filter with frequency cut-offs at 0.03 Hz and 14 Hz. For each complex and each pure tone (corresponding to the missing fundamental), the same five source and five sink channels from the magnetic contour map that provided the strongest detected signal were selected from each hemisphere (20 total channels). M100 latency was defined as the root-mean-square (RMS) peak across these channels within a post-stimulus window of 90–180 ms and recorded, along with field strength (measured in fT), for each stimulus type. A 70 ms burst of broadband noise was presented as part of a distracter task. The noise burst was presented independently, occurring 150 times at pseudo-random intervals over five blocks of approximately 9 minutes.

## Results

**Figure 2:**
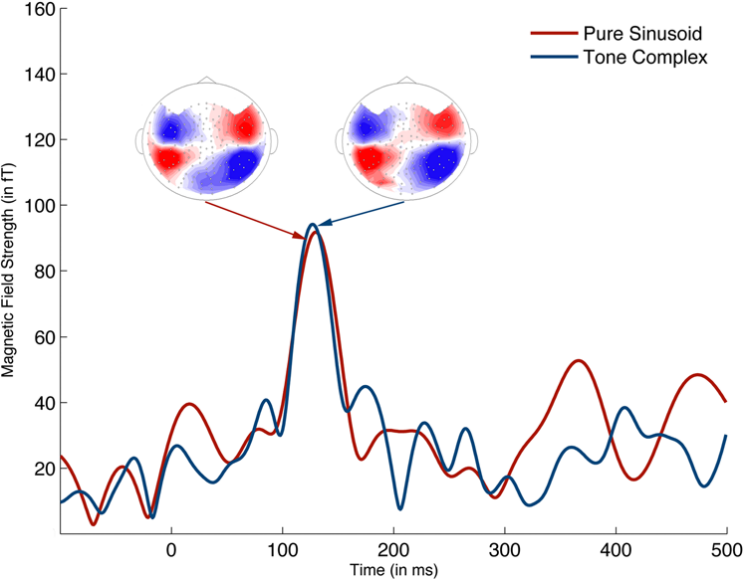
Comparison of the MEG waveforms to a pure sinusoid (in this case, 600 Hz) and tone complex with the corresponding inferred fundamental (in this case, 12-18-24) for a representative subject. Data is the RMS from 10 channels (five sink, five source) in the left hemisphere. The peak around 100 ms post-onset of the target (0 ms represents the onset of the target) is the M100. The peak latency of the M100 to the pure sinusoid and its corresponding tone complex were closely matched. The head-models represent the magnetic field contours for the M100. The red regions represent the source of the dipole and the blue regions represent the sink of the dipole.

**Figure 3:**
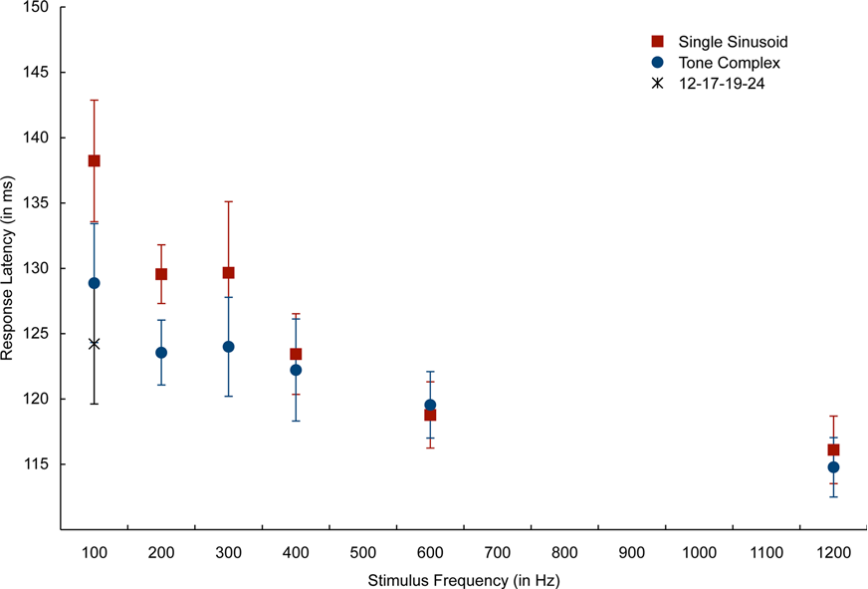
M100 RMS latencies to single sinusoid tones, tone complexes (plotted by their inferred fundamental component), and the 12-17-19-24 kHz tone complex, whose fundamental component is 100 Hz. Error bars refer to ±1 standard error of the group mean.

Figure 2 illustrates the RMS of a typical neuromagnetic response to both the pure sinusoid and its corresponding tone complex. Figure 3 shows mean M100 latency as a function of the fundamental frequency or missing fundamental. Statistical analyses were done using mixed-effects ANOVAs with Subject as a random effect, excluding the 12-17-19-24 complex tone to maintain a balanced design. Analysis of the latencies of the M100 responses showed main effects of frequency (*F*(5,88) = 11.15; *p*<0.0001) and signal type (pure sinusoid vs. tone complex; *F*(1,88) = 6.00; *p* = 0.016), but crucially, there was no interaction between signal type and frequency (*F*(5,88) = 1.02; *p* = 0.41). In planned post-hoc comparisons, we found no significant differences at each frequency between the M100 latency to the pure sinusoid and the M100 latency to the tone complex, though the difference between the M100 response latency to the 100 Hz sinusoid and the 100 Hz inferred pitch tone complex (12-13-23-24) was marginally significant (t(8) = 2.48; *p* = 0.015, n.s. due to multiple-comparisons correction, all others p>0.12). Analysis of the M100 amplitudes revealed a weakly significant main effect of frequency in which higher frequencies have larger amplitudes (*F*(5,88) = 2.79; *p* = 0.022), no main effect for signal type (*F*(1,88) = 0.54; *p* = 0.46), and a significant interaction between frequency and signal type (*F*(5,88) = 5.82; *p*<0.0001). Post-hoc comparisons (Tukey-Kramer honestly significant differences) found only one significant contrast: the amplitude of the sinusoidal 100 Hz response is significantly weaker than the amplitude of the sinusoidal 1200 Hz response. The significant interaction effect is due to a cross-over between the sinusoidal responses (which have increasing amplitudes with increasing frequency) and a generally level amplitude response to all of the tone complexes.

On a model that supposes that the M100 reflects just the physical properties of the stimulus, we would expect that the latencies to all tone complexes to be around 115 ms (the latency of the M100 to the 12–24 tone complex). In other words, we would anticipate that the 1200 Hz component present in each tone complex to drive a considerably faster M100 latency. This, however, is not the case. Instead, our findings suggest that the M100 is reflecting contributions of the inferred pitch of the stimulus and not solely the surface properties of the stimulus.

## Discussion

Using stimuli that incorporate a specific improvement over earlier materials, we replicated the M100 latency curve previously found [15]. Moreover, we found no latency difference between M100 responses to pure sinusoids versus tone complexes across frequencies. Our findings suggest that listeners are reconstructing the inferred pitch by roughly 100 ms after stimulus onset and are consistent with previous electrophysiological research suggesting that the inferential pitch is perceived in early auditory cortex [11]–[14]. Moreover, the nature of the stimuli in the present study suggest that it is not necessary for a tone complex to be comprised of adjacent harmonics for pitch to be inferred (cf., [13]).

These results provide information about the relative timing of when listeners reconstruct inferred pitch. In other words, whatever computations are germane to inferred pitch must be carried out in the initial stages of auditory processing. Understanding the time course of the perception of inferred pitch helps us to delimit the types of neurobiological computations involved. These findings do not allow us to decide between differing models of inferential pitch, but they do suggest that any model of pitch perception must place this reconstruction effect early in auditory processing. This conclusion is consistent with recent modeling research that proposes sub-cortical involvement in the reconstruction of virtual pitch via coordinated processing in populations of neurons [20], which is what MEG measures. Research on the integration time of the M100 shows that the M100 integrates over the first 40 ms of signal [21]–[23]; therefore the computations we are seeing here must be executed over no more than that amount of input (see Chait, *et al.* [24] for discussion of the spatial and temporal dynamics of pitch perception using MEG).

In addition to new information about inferred pitch, this study yields further insight into the nature of the M100 response itself. M100 latencies recorded in this study have been shown to co-vary with stimulus frequency when the stimuli were pure sinusoids, just as they were in Roberts and Poeppel [15]; but they have also been shown to vary with the inferred fundamentals of tone complexes. It is possible, then, to build on the findings in Roberts and Poeppel [15] and conclude that the M100 reflects computations that are performed over the whole spectrum of the signal, and not simply an index of the transparent spectral properties of a stimulus.

### Conclusion

MEG results suggest that listeners reconstruct the fundamental component of a complex tone early in auditory perception. In particular, by the time the neural generators of the M100 have been activated, we find evidence that listeners have reconstructed the fundamental component, indicating that some amount of abstract computations have been performed, in this case, the restoration of the fundamental component, early in auditory perception.
